# Retraction statement: Hu X‐M, Xiang J‐J, Xiao B‐L, Huang Y‐F and Xie J‐P (2019) Wogonoside promotes apoptosis in gastric cancer AGS and SGC‐7901 cells through induction of mitochondrial dysfunction and endoplasmic reticulum stress. *FEBS Open Bio* 9, 1469–1476.

**DOI:** 10.1002/2211-5463.13584

**Published:** 2023-03-17

https://doi.org/10.1002/2211-5463.12693.

The above article, published online on 28 June 2019 in Wiley Online Library (wileyonlinelibrary.com), has been retracted by agreement between the Editor‐in‐Chief, FEBS Press, and John Wiley and Sons Ltd. The retraction has been agreed following an investigation into concerns raised by a third party, which revealed inappropriate duplications between this and previously published articles [1, 2]. Thus, the editors consider the conclusions of this manuscript substantially compromised.
