# Correction

**DOI:** 10.1002/2211-5463.13928

**Published:** 2024-11-21

## Abstract

**CORRECTION: RETRACTION:** Wogonoside Promotes Apoptosis in Gastric Cancer AGS and SGC‐7901 Cells Through Induction of Mitochondrial Dysfunction and Endoplasmic Reticulum Stress

X.M. Hu, J.J. Xiang, B.L. Xiao, Y.F. Huang, and J.P. Xie, “Retraction statement: Hu X‐M, Xiang J‐J, Xiao B‐L, Huang Y‐F and Xie J‐P (2019) Wogonoside promotes apoptosis in gastric cancer AGS and SGC‐7901 cells through induction of mitochondrial dysfunction and endoplasmic reticulum stress,” *FEBS Open Bio*, no. 13 (2023): 796, https://doi.org/10.1002/2211-5463.13584.

Information regarding the coauthor Ying‐Fei Huang's non‐involvement with the research and writing of this article was erroneously missed in the above retraction notice and has been included in this version.

The corrected retraction notice is:

The above article, published online on 28 June 2019 in Wiley Online Library (wileyonlinelibrary.com), has been retracted by agreement between the Editor‐in‐Chief, FEBS Press, and John Wiley and Sons Ltd. The retraction has been agreed following an investigation into concerns raised by a third party, which revealed inappropriate duplications between this and previously published articles [[1, 2]]. Thus, the editors consider the conclusions of this manuscript substantially compromised.

Additionally, the coauthor Ying‐Fei Huang's institution University of New South Wales informed the journal that Ms. Huang was not involved with the research or writing of this article, and that her name and affiliation were used without her knowledge or permission.

References

1 Sun L, Jiang C, Xu C, Xue H, Zhou H, Gu L, Liu Y and Xu Q (2017) Down‐regulation of long non‐coding RNA RP11‐708H21.4 is associated with poor prognosis for colorectal cancer and promotes tumorigenesis through regulating AKT/mTOR pathway. *Oncotarget* **8**, 27929–27942.

2 Li Y, Tao C, Dai L, Cui C, Chen C, Wu H, Wei Q and Zhou X (2019) MicroRNA‐625 inhibits cell invasion and epithelial–mesenchymal transition by targeting SOX4 in laryngeal squamous cell carcinoma. *Biosci Rep* **39**, BSR20181882.

**CORRECTION: RETRACTION:** Wogonoside Promotes Apoptosis in Gastric Cancer AGS and SGC‐7901 Cells Through Induction of Mitochondrial Dysfunction and Endoplasmic Reticulum Stress

X.M. Hu, J.J. Xiang, B.L. Xiao, Y.F. Huang, and J.P. Xie, “Retraction statement: Hu X‐M, Xiang J‐J, Xiao B‐L, Huang Y‐F and Xie J‐P (2019) Wogonoside promotes apoptosis in gastric cancer AGS and SGC‐7901 cells through induction of mitochondrial dysfunction and endoplasmic reticulum stress,” *FEBS Open Bio*, no. 13 (2023): 796, https://doi.org/10.1002/2211-5463.13584.

Information regarding the coauthor Ying‐Fei Huang's non‐involvement with the research and writing of this article was erroneously missed in the above retraction notice and has been included in this version.

The corrected retraction notice is:

The above article, published online on 28 June 2019 in Wiley Online Library (wileyonlinelibrary.com), has been retracted by agreement between the Editor‐in‐Chief, FEBS Press, and John Wiley and Sons Ltd. The retraction has been agreed following an investigation into concerns raised by a third party, which revealed inappropriate duplications between this and previously published articles [[1, 2]]. Thus, the editors consider the conclusions of this manuscript substantially compromised.

Additionally, the coauthor Ying‐Fei Huang's institution University of New South Wales informed the journal that Ms. Huang was not involved with the research or writing of this article, and that her name and affiliation were used without her knowledge or permission.

**References**

1 Sun L, Jiang C, Xu C, Xue H, Zhou H, Gu L, Liu Y and Xu Q (2017) Down‐regulation of long non‐coding RNA RP11‐708H21.4 is associated with poor prognosis for colorectal cancer and promotes tumorigenesis through regulating AKT/mTOR pathway. *Oncotarget* **8**, 27929–27942.

2 Li Y, Tao C, Dai L, Cui C, Chen C, Wu H, Wei Q and Zhou X (2019) MicroRNA‐625 inhibits cell invasion and epithelial–mesenchymal transition by targeting SOX4 in laryngeal squamous cell carcinoma. *Biosci Rep* **39**, BSR20181882.

